# Systemic versus topical administration of antioxidants in radiation-induced oral mucositis

**DOI:** 10.6026/973206300212149

**Published:** 2025-07-31

**Authors:** Saikiran Bahadur, Diksha Koushal, Eesha, Rajeeth Shetty, Alpana Kanwar, Rahul Kumar Tomar, Sitesh Kumar Samal

**Affiliations:** 1 Departments of Prosthodontics, Springfield Dental, Springfield, Massachussets, United State of America; 2 Department of Oral Medicine and Radiology, Jaipur Dental College, Jaipur, Rajasthan, India; 3 Department of Oral Pathology & Microbiology, Regional Dental College (RDC), Guwahati, India; 4 Department of Oral and Maxillofacial Surgery, Srinivas Institute of Dental Sciences, Mukka, Mangalore, Karnataka, India; 5 Department of Oral and Maxillofacial Pathology & Microbiology, Maharaja Ganga Singh Dental College and Research Centre, Sri Ganga Nagar, Rajasthan, India; 6 Dapartment of Oral Pathology and Microbiology, DJ College of Dental Sciences and Research, Modinagar, Ghaziabad, Uttar Pradesh, India; 7 Intern, Kalinga Institute of Dental Science, Kalinga Institute of Industrial Technology (KIIT) Deemed to be University, Patia, Bhubaneswar, Odisha, India

**Keywords:** Oral mucositis, antioxidants, radiation therapy, Vitamin E, curcumin gel

## Abstract

Oral mucositis (OM) is a debilitating complication of radiotherapy in head and neck cancer patients. This study was done to compare the efficacy of systemic versus topical antioxidant therapy in preventing and managing radiation-induced oral mucositis. Total 150 head and neck cancer patients undergoing radiotherapy were divided into systemic antioxidants and topical antioxidants groups. Further subdivide into three subgroups based on the antioxidant agent used. Clinical parameters were assessed using the WHO oral mucositis scale. Both systemic and topical antioxidants reduced the severity of OM; however, topical antioxidants, especially curcumin gel, showed superior efficacy.

## Background:

Oral mucositis (OM) remains one of the most distressing complications of radiotherapy and chemo-radiotherapy in patients with head and neck cancers, affecting nearly 80% of individuals undergoing treatment [1, 2]. It presents with erythema, painful ulcerations and significant discomfort, leading to impaired oral intake, speech difficulties, weight loss and even treatment delays, which can compromise overall prognosis [3, 4]. The pathogenesis of OM is a multifactorial process that begins with oxidative stress and the excessive production of reactive oxygen species (ROS), resulting in direct DNA damage, inflammatory cascade activation and eventual mucosal breakdown [5, 6, 7-8]. Strategies to manage OM have evolved from symptomatic care to targeted interventions aimed at modifying underlying biological processes. Antioxidants have gained attention as promising agents due to their ability to neutralize ROS and attenuate the inflammatory response, thereby potentially reducing mucosal injury and accelerating healing [6, 8 and 9]. While systemic administration of antioxidants offers widespread protection against oxidative damage, topical delivery provides direct therapeutic action at the mucosal surface, potentially enhancing local bioavailability and minimizing systemic side effects [7]. Recent meta-analyses and clinical guidelines suggest both routes are effective but emphasize the need for comparative clinical studies to determine optimal strategies for OM prevention and management [1, 2 and 5]. Therefore, it is of interest to evaluate and compare the clinical efficacy of systemic and topical antioxidant therapies in mitigating radiation-induced oral mucositis in head and neck cancer patients. By assessing multiple agents in each category, this investigation seeks to provide evidence-based insights into their relative benefits, thus guiding clinicians toward more effective and patient-centred management of OM.

## Materials and Methods:

This prospective, randomized comparative study was conducted on 150 patients with head and neck cancers scheduled to receive ≥50 Gy of radiotherapy. Participants aged between 18 and 70 years were enrolled after meeting inclusion criteria, which required a confirmed diagnosis of head and neck malignancy and exclusion of those with pre-existing oral lesions, immunosuppressive conditions, or prior antioxidant therapy. Patients were randomly assigned to two main groups: systemic antioxidant therapy (n=75) and topical antioxidant therapy (n=75). The systemic group was further divided into three subgroups: Group S1 received Vitamin E 400 IU daily, Group S2 received N-acetylcysteine (NAC) 600 mg daily, and Group S3 received Lycopene 10 mg daily, each with 25 patients. Similarly, the topical group was subdivided into Group T1 (curcumin gel 0.5%, applied three times daily), Group T2 (aloe vera gel, applied three times daily), and Group T3 (Vitamin E mouthwash 0.1%, used three times daily), with 25 patients in each subgroup. Clinical assessments included WHO Oral Mucositis Grading performed at baseline and weekly for six weeks, visual analog scale (VAS) scores for pain, duration of mucositis (in days), and the need for opioid analgesics. Statistical analysis was carried out using SPSS version 26, employing t-tests, ANOVA, and Chi-square tests, with a p-value <0.05 considered statistically significant.

## Results:

A total of 150 patients were enrolled and equally distributed between the systemic (n=75) and topical (n=75) antioxidant therapy groups. The demographic characteristics of both groups were comparable, with no statistically significant differences in mean age (52.4 ± 8.7 years vs. 51.8 ± 7.9 years; p = 0.621), gender distribution (Male: Female ratio 43:32 vs. 41:34; p = 0.817), or mean radiation dose received (62.5 ± 4.2 Gy vs. 62.8 ± 3.9 Gy; p = 0.743) (Table 1 - see PDF). Both groups had a baseline mucositis score of 0 before initiating radiotherapy. At the end of six weeks, the topical antioxidant group demonstrated significantly lower peak mucositis grades compared to the systemic group (mean WHO grade 1.7 ± 0.4 vs. 2.3 ± 0.5; p = 0.001). Additionally, the mean duration of mucositis was markedly shorter in the topical group (8.5 ± 2.7 days) than in the systemic group (11.2 ± 3.1 days; p = 0.004). The requirement for opioid analgesics was also significantly reduced in the topical group (30%) compared to the systemic group (48%; p = 0.028). Among individual antioxidants, topical curcumin gel (T1) demonstrated the greatest efficacy, with the lowest peak mucositis grade (mean 1.1 ± 0.3). In the systemic group, Vitamin E (S1) was the most effective, showing a lower mucositis grade relative to N-acetylcysteine and Lycopene subgroups.

## Discussion:

Oral mucositis (OM) remains a significant dose-limiting toxicity in patients receiving radiotherapy or chemoradio-therapy for head and neck cancers, with an incidence of up to 80% depending on treatment intensity and modality [7]. The multifactorial pathogenesis of OM, as described by Sonis [8] (2004), involves five overlapping stages, beginning with DNA damage and reactive oxygen species (ROS) generation, leading to pro-inflammatory cytokine release and culminating in ulceration and impaired healing. ROS play a pivotal role as both initiators and amplifiers of mucosal injury, affecting cellular survival, apoptosis and senescence pathways [10]. This study demonstrated a significant reduction in peak mucositis grade and duration with topical antioxidant therapy compared to systemic administration. Notably, topical curcumin gel achieved the lowest mean mucositis grade among all groups, suggesting its potent local anti-inflammatory and antioxidant effects. Curcumin and its derivatives are known to scavenge free radicals and modulate NF-κB and COX-2 pathways, which are central to mucosal injury progression [11]. This finding aligns with prior evidence supporting the efficacy of plant-derived antioxidants for OM management [1]. Systemic Vitamin E, the most effective agent in our systemic group, exhibited a moderate reduction in mucositis severity. Its lipid-soluble properties and ROS-quenching ability support its role in mitigating oxidative damage; however, its systemic administration may result in suboptimal concentrations at the mucosal surface [12, 13]. These observations support the concept that local delivery of antioxidants may provide superior mucosal bioavailability and therapeutic efficacy, a notion echoed in MASCC/ISOO guidelines [14]. Furthermore, the observed decrease in opioid analgesic use among patients receiving topical antioxidants underscores the clinical relevance of improved mucositis management. Effective control of OM not only enhances patient comfort but also helps maintain nutritional intake and reduces the risk of treatment interruptions, which is critical in oncologic outcomes. Despite promising results, concerns have been raised regarding the concurrent use of antioxidants during cancer therapy, given their potential to interfere with ROS-mediated tumor cytotoxicity [12]. However, recent reviews and meta-analyses suggest that antioxidants, when judiciously used, may protect normal tissues without compromising tumor response [13]. The strength of our study lies in its comparative evaluation of multiple systemic and topical agents with a randomized design. However, the relatively small sample size and single-center nature limit generalizability. Additionally, longer follow-up is needed to assess potential effects on tumor control. Further multi-center, large-scale randomized controlled trials are recommended to validate these findings. Investigations combining systemic and topical antioxidant regimens may also reveal synergistic effects in OM prevention and management. Small sample size; single-center studies are limitations of the study. Multi-center trials with larger cohorts and combined systemic-topical regimens are warranted.

## Conclusion:

This study highlights the potential of antioxidant therapy in reducing the severity and duration of radiation-induced oral mucositis in head and neck cancer patients. Both systemic and topical antioxidants were effective; however, topical agents, particularly curcumin gel, demonstrated superior efficacy in minimizing mucosal injury, pain and opioid analgesic requirements. These findings emphasize the importance of localized antioxidant delivery for better therapeutic outcomes and patient comfort during radiotherapy.

## References

[1] Raza A (2022). Support Care Cancer..

[2] Chaitanya N.C (2017). J Clin Diagn Res..

[3] Lalla R.V (2008). Dent Clin North Am..

[4] https://www.oralhealthgroup.com/.

[5] Lalla R.V (2014). Cancer..

[6] Villa A, Sonis ST. (2016). Expert Opin Pharmacother..

[7] Raber-Durlacher J.E (2010). Oral Oncol..

[8] Sonis S.T (2004). Nat Rev Cancer..

[9] Basile D (2019). Cancers (Basel)..

[10] Panieri E (2013). Free Radic Biol Med..

[11] Sahu P.K (2016). Bioorganic Med Chem Lett..

[12] Block K.I (2007). Cancer Treat Rev..

[13] Worthington H.V (2011). Cochrane Database Syst Rev..

[14] Elad S (2020). Cancer..

